# Role of early childhood educators’ demographic characteristics and perceived work environment in implementation of a preschool health promotion intervention

**DOI:** 10.1186/s13690-023-01133-z

**Published:** 2023-07-07

**Authors:** Jasmine Gustafsson, Carola Ray, Elviira Lehto, Eva Roos, Reetta Lehto

**Affiliations:** 1 Public Health Research Program, Folkhälsan Research Center, Helsinki, Finland; 2 Faculty of Social Sciences, University of Helsinki, Helsinki, Finland; 3 Department of Food and Nutrition, University of Helsinki, Helsinki, Finland; 4 Department of Teacher Education, University of Helsinki, Helsinki, Finland; 5 Department of Food Studies, Nutrition and Dietetics, Uppsala University, Uppsala, Sweden; 6 Department of Public Health, University of Helsinki, Helsinki, Finland

**Keywords:** Preschool, Implementation, Fidelity, Health intervention, Work environment

## Abstract

**Background:**

Research has indicated that the effectiveness of intervention programs is affected by how well these programs are implemented, but key gaps remain in our understanding of the factors that promote or inhibit implementation. This study examined how demographic characteristics and perceived work environment among early childhood educators were associated with implementation outcomes of the Increased Health and Wellbeing in Preschools (DAGIS) intervention, which was conducted as a cluster randomized trial.

**Methods:**

Participants included 101 educators from 32 intervention preschool classrooms. Data were analyzed at the classroom level, as the DAGIS intervention was delivered in preschool classrooms consisting of several educators instead of individual implementers. Linear regression was used to estimate the associations of educators’ demographic characteristics and perceived work environment with different aspects of implementation (i.e., dose delivered; dose received – exposure; dose received – satisfaction; and perceived quality, as well as a total sum score based on these four dimensions). Municipality was controlled in the adjusted models.

**Results:**

Findings indicated that having a higher proportion of educators with a Bachelor’s or Master’s degree in education within the classroom was associated with higher dose received – exposure and higher total degree of implementation, and the significance of the models was unaffected by adjustment for municipality. Moreover, having a higher proportion of educators younger than 35 years within the classroom was associated with higher dose received – exposure. However, the association was non-significant when adjusted for municipality. No other educator factor (i.e., work experience in years and perceived support from coworkers, group work, and innovative climate) predicted implementation outcomes.

**Conclusions:**

Higher educational attainment and younger age among educators at the classroom level were associated with higher scores for some of the implementation outcomes. Educators’ work experience in years at the current preschool and in early childhood education, support from coworkers, group work, and innovative climate were not significantly associated with any implementation outcomes. Future research should explore ways to improve educators’ implementation of interventions aimed at promoting children’s health behaviors.

**Supplementary Information:**

The online version contains supplementary material available at 10.1186/s13690-023-01133-z.

**Table:** 

**Text box 1. Contributions to the literature**
• Research has shown that only a small portion of the health interventions delivered in preschools have beneficial effects, which might partially be due to the fact that educators do not necessarily implement intervention programs as intended.
• We found evidence that suggests that some of the educators’ demographic characteristics (i.e., higher educational attainment and younger age) was linked to some aspects of implementation, mainly the program activities implemented directly by the educators.
• Our findings may be used to improve implementation and, ultimately, intervention efficacy by identifying educators who may need additional support to implement these types of programs.

## Background

Childhood obesity remains an important public health concern since obesity rates have increased worldwide among children from 1975 to 2016 [1]. Among children, the most important behavioral determinants of being overweight include unfavorable energy balance-related behaviors (EBRB) such as low levels of physical activity, high levels of sedentary behavior, and consumption of energy-dense foods [2]. Moreover, obesity is assumed to be predicted by ineffective self-regulation skills combined with high stress levels among children (for review, see Aparicio et al. [3]).

The preschool setting appears to be propitious for interventions to promote healthy child EBRB [4] and self-regulation skills (for review, see Savina [5]). However, only a small portion of the interventions performed are shown to have beneficial effects [6], which might partially be due to low implementation levels, as teachers do not necessarily implement intervention programs as intended. For example, Becker et al. [7] found that only 55% of teachers achieved high levels of implementation in a game intervention conducted in preschools and elementary schools. In line with these findings, teachers scored on average only slightly more than half of the total implementation score in a preschool health intervention [8]. This is concerning, as a key aspect of effective interventions is that the program is put into practice as intended [9]. To achieve greater implementation success, research must explore which factors facilitate or hinder implementation of interventions in the preschool setting. Furthermore, it is important to identify staff members in need of additional support and encouragement to implement these programs.

### Dimensions of implementation

Implementation commonly refers to what an intervention program comprises when it is delivered in a specific setting, and evaluating implementation has been noted as important for measuring the internal and external validity of interventions [10]. Several frameworks and models to guide the assessment of implementation have been developed, with partially overlapping dimensions. For example, Durlak and DuPre [10] suggested that there are eight aspects of implementation: fidelity, which describes the degree to which the intervention corresponds to the originally intended program; dosage, referring to how much of the original program has been delivered; quality, which includes how well different components of the program are delivered; participant responsiveness, which describes to what extent the program evokes interest of intervention participants (e.g., children); program differentiation, referring to what extent a program’s theory and practices are distinct or unique from other programs; monitoring of control/comparison conditions, which describes the types and amount of program content received by participants of these groups; program reach, which includes the proportion of participants who participated in the intervention of those who were qualified to receive the program; and adaptation, referring to the extent to which the original program can be modified during implementation (e.g., to fit the needs of the providers of the program).

To assess implementation of health-promoting interventions specifically, Saunders et al. [11] developed a process-evaluation plan that comprises five elements: fidelity (quality), dose delivered, dose received – exposure, dose received – satisfaction, reach, recruitment, and context. In their process-evaluation plan, fidelity refers to the quality of the intervention, that is, to what extent the program was implemented as planned. Dose delivered refers to the degree to which the intended units or components of the intervention were delivered by the interventionists. Dose received – exposure is defined as the degree to which participants actively engage with, use, or receive intervention materials or resources. Dose received – satisfaction refers to the extent to which participants are satisfied with the intervention program. Reach refers to quantifying how much of the intended target audience took part in the intervention. Recruitment includes procedures related to recruiting and approaching participants, and context refers to contextual aspects that affect implementation or intervention outcomes.

### Teacher characteristics and implementation

Previous implementation research has examined whether teachers’ implementation of programs was associated with different demographic characteristics of the implementers, with most studies conducted in an elementary school context. The findings have been mixed. For example, higher implementation levels have been associated with teacher factors such as younger age [12], being female [13], higher educational level [14], having teacher certification [15], being educated in fields other than early childhood education or elementary education [16], and having less teaching experience [17]. Other studies have yielded the opposite results, as better program implementation has been linked to older age among teachers [18] and having greater teaching experience [19]. Still other studies did not find teachers’ age [20], sex [12, 20], educational attainment [12, 21], or years of teaching experience [14, 16, 18, 20, 21] to be related to any components of implementation.

The degree of implementation has also been suggested to be influenced by teachers’ perceptions of the work environment. Previous studies have identified a link between higher implementation levels and teachers’ perceptions of having supportive colleagues [22], a supportive school climate [23], a positive organizational climate [14], a positive innovative climate [22], a positive work environment [21], greater connectedness with the school [24], and higher ratings of organizational health (e.g., teacher affiliation and collegial leadership) [20]. On the other hand, one study found that teachers’ organizational climate (involving e.g., perceptions of teamwork) was negatively related to implementation quality [14], and insignificant associations between implementers’ work environment and implementation outcomes have been observed as well (e.g., [12, 19, 25]).

In sum, several previous studies, most of them conducted in the United States, have examined whether teacher characteristics contribute to achieved implementation levels in the educational setting. However, the evidence has been mixed, which could be due to different contextual factors, such as variations in intervention outcome, length of intervention, program content, and number of activities to be delivered by teachers. Maybe most importantly, implementation outcomes have been defined in different ways and assessed by several different measures, making it difficult to compare findings across studies [26]. Thus, several questions remain about the role of implementer predictors of intervention implementation, and further research guided by implementation frameworks is warranted to clarify the mechanisms underlying the variations in implementation levels in preschool settings. In addition, there is a growing need for a more comprehensive evaluation of implementation of interventions targeting children’s EBRBs [6]. In this study, the following research question was addressed: how were educators’ demographic characteristics (educational attainment, age, and work experience in years) and perceived work environment (support from coworkers, group work, and innovative climate) associated with implementation outcomes of the Increased Health and Wellbeing in Preschools (DAGIS) intervention in preschool classrooms? The main aim of the DAGIS intervention, which was applied in Finland, was to promote healthy EBRBs and self-regulation skills among 3–6-year-old children [27]. Following the process-evaluation plan developed by Saunders et al. [11], four aspects of educators’ implementation of DAGIS in preschools were examined: 1) dose delivered, 2) dose received – exposure, 3) dose received – satisfaction, and 4) perceived quality, as well as the total sum score based on these four dimensions, i.e., the total degree of implementation. In this study, the term “educator” is used to describe the preschool personnel, regardless of whether they had an early childhood teaching qualification. In contrast to most previous implementation studies in the field, data were only analyzed at the classroom level, as the DAGIS intervention was delivered in preschool classrooms consisting of several educators.

## Method

### DAGIS intervention study

The DAGIS intervention study was conducted as a cluster randomized trial designed to promote 3–6-year-old children’s healthy EBRBs and self-regulation skills. The intervention was planned using the Intervention Mapping (IM) protocol [28], and the development process has been described elsewhere [27]. The DAGIS intervention was conducted during the school year from September 2017 to May 2018 and included baseline and follow-up measurements. The study was conducted in two municipalities in Southern Finland. In total, 32 preschools and 802 children participated. After baseline measurements, the preschools were randomized into 13 intervention and 19 control preschools. Further details of the recruitment process have been provided elsewhere [29]. Given the focus on implementation outcomes in the current study, only intervention preschools were considered, and control preschools were excluded. The intervention program involved families as well. In this study, only activities implemented in preschools were considered (i.e., not activities implemented by parents or caregivers). The Helsinki Ethics Review Board in Humanities and Social and Behavioral Sciences approved the collection and treatment of all data for the project (22/2017; 16 May 2017).

### DAGIS program content

In intervention preschools, all educators were invited to participate in two program training sessions: a longer training session lasting approximately 5 h at the beginning of the intervention and a shorter training session lasting approximately 3 h around the middle of the 23-week program. Training sessions were held by two persons: a researcher in the field of children’s EBRBs and a project coordinator with a Master’s degree in education as well as teaching experience in early childhood education. During the sessions the educators received the instructions and the required materials for the program activities. Throughout the intervention, educators also received pedagogical booster e-mails as a reminder of each theme period that was going to begin. The e-mails also included additional practical tips for how the themes could be discussed with children. Educators were able to contact the research team by e-mail in case they had further questions during the intervention.

The DAGIS intervention program consisted of five main theme periods: strengthening self-regulation skills, enhancing physical activity, promoting consumption of fruits and vegetables, reducing excessive screen time, and restricting the consumption of sugary foods and beverages. Each theme period lasted 4 to 5 weeks. The intervention was largely based on the international MindUp™ program [30]. The MindUp™ program included short relaxation moments for children, which were intended to be conducted three times daily. Moreover, the MindUp™ program involved EBRB-promoting strategies, which were added to the DAGIS intervention. The DAGIS program also included other educational activities for children: for instance, one storybook about balancing screen time and physical activity, a second storybook about being brave with tasting food, discussions on the program themes with the help of fictional animal characters, sensory-based activities related to fruits and vegetables, and games to increase physical activity. For each theme, preschools were also asked to arrange an activity afternoon (i.e., a workshop to which children and their parents were invited). The materials have been described more comprehensively elsewhere [27].

### Data collection and participants

Data were collected from September 2017 to April–May 2018. Data collection consisted of two educator questionnaires as well as several questionnaires and logbooks completed by educators or contact persons, as one educator from each classroom had been assigned the role of contact person. Predictor variables were measured at the beginning of the intervention using a questionnaire completed by educators, whereas implementation variables were assessed during the intervention through several questionnaires and logbooks completed by educators or contact persons.

Participants included 145 educators from 45 classrooms in 13 intervention preschools. Three classrooms did not complete any educator questionnaires and were thus not included in this study. Moreover, only one educator provided data in 10 classrooms, which prevented data aggregation, resulting in a final sample of 32 classrooms in 11 preschools located in two different municipalities (Salo, *n* = 25; Riihimäki, *n* = 7). A final sample of 101 educators was included in this study.

Educators were asked to complete two educator questionnaires. The first educator survey, collected at the beginning of the intervention, was completed by 90 educators (62% response rate). The second educator survey, collected at the end of the intervention, was completed by 86 educators (59% response rate). Of the educators who answered the second survey, 15 respondents had not completed the first questionnaire but were still included in this study. Data were available for two to six educators in each classroom. Within 10 classrooms (31%), data were available for two educators, within 10 classrooms (31%) for three educators, within 10 classrooms (31%) for four educators, within 1 classroom (3%) for five educators, and within 1 classroom (3%) for six educators.

### Measures

#### Predictor variables

##### Educators’ demographic characteristics

Information on educators’ sex, year of birth (age), educational attainment, and work experience in years at the current preschool and in early childhood education was collected. Given that only 2% of the educators who answered the questionnaires were men, it was not possible to analyze sex as a potential predictor of implementation outcomes. As the demographic characteristics were assessed at individual level and most implementation outcomes were assessed at classroom level, measures of multiple individuals within the same classroom were aggregated to the classroom level. For educators’ age and work experience in years at the current preschool and in early childhood education, individual responses were aggregated into group variables, describing the proportion (%) of educators within each classroom being younger than 35 years, having less than 5 years of experience at the current preschool, and having less than 10 years of experience in early childhood education. These cut-off points were selected based on previous research, given that younger age and having less work experience have been associated with higher implementation levels in previous studies [12, 17]. Educators’ educational attainment was assessed by asking one question about the highest formal education in early childhood education, with eight response categories (1 = none, 2 = other, which education?, 3 = vocational qualification in social and health care, 4 = Bachelor of social services (i.e., polytechnic diploma), 5 = college-educated social pedagogue/educator, 6 = college-educated kindergarten teacher, 7 = Bachelor’s degree in education, 8 = Master’s degree in education). Open-ended responses to this question were also considered (i.e., polytechnic diplomas and vocational degrees). In Finland, vocational schools, also called trade schools, are institutions of secondary education, while polytechnics are institutions of tertiary education – both usually viewed as providers of practically oriented skills. Educators’ educational attainment was aggregated into a group variable, describing the proportion (%) of educators within each classroom having a Bachelor’s or Master’s degree in education.

##### Educators’ perceived work environment

Educators’ perceived work environment was assessed using three dimensions of the General Nordic Questionnaire for Psychological and Social Factors at Work (QPSNordic), which is a validated questionnaire designed to assess a comprehensive set of social and psychological aspects of the work environment [31]. The validation of the QPSNordic has been carried out in data collected from four Nordic countries, including Finland [31], and later in a separate sample of Swedish employees [32]. The following dimensions were assessed: support from coworkers (2 items), group work (3 items), and innovative climate (3 items). For the items included in these variables, see Additional file 1. Educators responded on a 5-point Likert scale, ranging from 1 = very seldom or never to 5 = very often or always. First, a mean score for each dimension at the individual level was calculated. Values of Cronbach’s alpha were as follows: support from coworkers = 0.66, group work = 0.78, and innovative climate = 0.45. For the final variables, the average rating for each dimension across the preschool classrooms was calculated. Intraclass correlation coefficients (ICCs) were support from coworkers = 0.37, group work = 0.42, and innovative climate = 0.29 (number of raters 2–6).

#### Implementation outcomes

##### Dose delivered

Dose delivered was measured by rating three elements: (1) whether preschool educators received the materials and e-mails from the researchers (i.e., e-mails not being bounced back rated as received), (2) the proportion of educators within each classroom who took part in the first training session, and (3) the proportion of educators within each classroom who took part in the second training session. Each item was scored separately and then calculated into a total score of dose delivered at the preschool classroom level (maximum 15). For the items and their scores, see Additional file 2.

##### Dose received – exposure

Dose received – exposure was assessed by asking the educators questions about whether or how often their classroom had implemented program activities in all five main themes of DAGIS. These questions were asked in logbooks for the whole classroom. The program activities included 64 items, which were scored separately. The scoring of individual items depended on how burdensome the item (e.g., activity) was expected to be for the educators. Next, the scores of these items were summed into a total sum score (maximum 59.8 points). To adjust the score in proportion to the other implementation outcomes, this score was multiplied by 0.75. Thus, the final score had a maximum score of 44.85. The maximum total score was the highest for this dimension, as it consisted of program activities delivered directly by the educators, which were expected to have the most impact on the behavior of the children. The items and their scores are presented in Additional file 3.

##### Dose received – satisfaction

Dose received – satisfaction was assessed by asking all educators 13 questions or to make statements about the extent to which they were satisfied with the intervention program. For each item, the average rating across the classrooms was calculated, with higher scores indicating higher satisfaction. Questions about overall satisfaction of the intervention were scored higher than those focusing on smaller subareas of satisfaction and specific themes of the program. For the final score, the average classroom ratings of all 13 items were added to a sum score (maximum 16), with ICC being 0.57 (number of raters 2–6). The items and their scores can be found in Additional file 4.

##### Perceived quality

Perceived quality was measured by asking all educators one question: overall, how well do you think your classroom delivered the DAGIS program (content and activities)? Again, responses were averaged across the classrooms and scored with a maximum of 15 points, with higher scores indicating higher perceived quality. ICC was 0.49 (number of raters 2–6). For the items and their scores, see Additional file 5.

##### Total degree of implementation

Based on the four aspects of educators’ implementation of DAGIS in preschools (dose delivered, dose received – exposure, dose received – satisfaction, and perceived quality), a total implementation score was computed, with a maximum score of 90.85.

#### Covariate

One covariate, i.e., municipality (1 = Salo, 2 = Riihimäki) was controlled, as the intervention preschools were located in two different municipalities.

### Analytic strategy

Statistical analyses were conducted using IBM SPSS Statistics 28.0. First, to enable assessment of all educators (*n* = 101), multiple imputation was performed for those who were missing information about perceived work environment in the first educator survey (*n* = 15 educators). Multiple imputation, which is one way of dealing with missing data, was performed assuming data were missing at random (MAR), and therefore could be predicted from the observed data [33]. Data were imputed by conducting a stepwise linear regression model using data from the second educator survey on these educators’ demographics (age, educational attainment, and work experience in years). Independent samples t-test revealed no significant differences in ratings of perceived work environment between observed and imputed values. No other missing values were imputed.

Descriptive statistics were derived from means (*M*), standard deviations (*SD*), and frequencies (%). In further analyses, measures of multiple individuals within the same classroom were aggregated to the classroom level, as most outcome variables were assessed at classroom level and all predictor variables were assessed at individual level. In addition, some of the implementation items (i.e., dose received – satisfaction and perceived quality) were aggregated to the classroom level. Pearson product-moment correlation test was used to examine possible associations between the key variables. As residuals and scatter plots revealed that assumptions of linearity, normality, and homoscedasticity were met reasonably well, linear regression was conducted to examine whether predictor variables were associated with implementation of the DAGIS intervention. First, each predictor’s relationship with each implementation outcome was assessed, and thereafter, significant predictors were entered into multivariate regressions. In the multivariate regressions, one predictor variable was tested, while controlling for municipality as a potential covariate. The small number of classrooms (*N* = 32) for the present sample was considered too low to include more than two independent variables in the multivariate models. *P*-values < .05 were considered statistically significant.

## Results

### Descriptive statistics of educators and correlations among study variables

Individual-level data on educators’ demographic characteristics and perceived work environment are presented in Table 1. Most educators were women (98%) and had a degree below Bachelor level (86%). At the classroom level, 12 of the 32 classrooms (38%) had one or more educators with a Bachelor’s or Master’s degree in education. In 7 of the 32 classrooms (22%), one or more educators within the classroom were younger than 35 years. The majority of the classrooms (26 of 32, 81%) had one or more educators with less than 5 years’ work experience at the current preschool, and half of the classrooms (16 of 32, 50%) had one or more educators with less than 10 years’ work experience in early childhood education. The means and standard deviations for classroom-level variables and their correlations are presented in Table 2. The results showed that among the predictor variables educators’ higher educational attainment correlated significantly and positively with implementation in terms of dose received – exposure and the total degree of implementation (*p* < .05). In addition, educators’ younger age correlated positively with dose received – exposure (*p* < .05).

**Table 1:** Individual-level descriptive characteristics of educators in the DAGIS intervention program, 2017–2018 (*n* = 97–101)

Variable	n (%)	M (SD)	Range
Sex			
Female	99 (98)		
Male	2 (2)		
Educational attainment			
Master^a^	1 (1)		
Bachelor^a^	13 (13)		
Polytechnic	14 (14)		
Vocational	72 (71)		
Below vocational	1 (1)		
Age (years)		47.02 (9.39)	19–62
Work experience (years)			
At current preschool		7.42 (8.14)	0–42
In early childhood education		17.55 (9.21)	0–41
Perceived work environment			
Support from coworkers		4.59 (0.55)	1–5
Group work		4.48 (0.55)	1–5
Innovative climate		3.65 (0.59)	1–5

^a^Master’s / Bachelor’s degree in education

**Table 2:** Classroom means, standard deviations, and bivariate correlations of classroom-level variables in the DAGIS intervention program, 2017–2018 (*N* = 32 preschool classrooms)

Variable	Classroom mean (SD)	1	2	3	4	5	6	7	8	9	10	11
1 Educational attainment^a^		-										
2 Age^b^		.32	-									
3 Work experience at current preschool^c^		.01	.00	-								
4 Work experience in early childhood education^d^		.37*	.38*	.47**	-							
5 Support from coworkers	4.57 (0.45)	.22	-.17	-.25	-.21	-						
6 Group work	4.46 (0.48)	.29	.11	-.32	-.06	.77**	-					
7 Innovative climate	3.66 (0.47)	.26	.05	-.44*	-.16	.52**	.40*	-				
8 Dose delivered	12.38 (1.77)	.16	.07	-.16	-.15	-.16	-.02	.01	-			
9 Dose received – exposure	23.67 (7.79)	.35*	.41*	.07	.11	-.08	.15	-.11	.09	-		
10 Dose received – satisfaction	10.42 (2.66)	.28	.09	-.29	-.28	.03	.06	.29	.37*	.38*	-	
11 Perceived quality	10.27 (1.90)	.00	-.11	-.08	-.14	-.05	.03	-.19	.02	.51**	.42*	-
12 Total degree of implementation	56.73 (10.76)	.35*	.31	-.06	-.04	-.09	.12	-.04	.33	.92**	.66**	.65**

Pearson product-moment correlation. Scores ranged from 1 to 5 for support from coworkers, 1 to 5 for group work, and 1 to 5 for innovative climate. Maximum values were 15 for dose delivered, 44.85 for dose received – exposure, 16 for dose received – satisfaction, 15 for perceived quality, and 90.85 for the total degree of implementation

^*^p < .05, ** *p* < .01

^a^Higher educational attainment (% of educators within classrooms with a Bachelor’s or Master’s degree in education)

^b^Younger age (% of educators aged less than 35 years within classrooms)

^c^Less work experience at current preschool (% of educators within classrooms having less than 5 years’ experience)

^d^Less work experience in early childhood education (% of educators within classrooms having less than 10 years’ experience)

### Predictors of implementation outcomes

Results from linear regressions for predictors and implementation outcomes are presented in Tables 3 and 4. Univariate analyses showed that educators’ educational attainment predicted dose received – exposure (B = 0.14, 95% Confidence Interval (CI) 0.00, 0.27) and the total degree of implementation positively (B = 0.19, 95% CI 0.00, 0.37); having a higher proportion of educators with a Bachelor’s or Master’s degree in education within the classroom was associated with higher dose received – exposure and higher total degree of implementation. Moreover, educators’ younger age predicted dose received – exposure positively (B = 0.23, 95% CI 0.04, 0.42), that is, having a higher proportion of educators younger than 35 years within the classroom was associated with higher dose received – exposure. Educators’ work experience in years at the current preschool and in early childhood education, and perceptions of support from coworkers, group work, and innovative climate were not significantly associated with any implementation outcomes.

**Table 3:** Results from the linear regression analyses showing univariate associations for each implementation outcome variable in the DAGIS intervention program, 2017–2018 (*N* = 32 preschool groups)

Variable	Dose delivered				Dose received - exposure				Dose received - satisfaction				Perceived quality				Total degree of implementation			
	B (β)	SE B	(95% CI for B)	*R*^2^	B (β)	SE B	(95% CI for B)	*R*^2^	B (β)	SE B	(95% CI for B)	*R*^2^	B (β)	SE B	(95% CI for B)	*R*^2^	B (β)	SE B	(95% CI for B)	*R*^2^
Educational attainment^a^	0.01 (0.16)	0.02	(-0.02, 0.05)	.03	0.14 (0.35)*	0.07	(0.00, 0.27)	.12	0.04 (0.28)	0.02	(-0.01, 0.08)	.08	-0.00 (-0.00)	0.02	(-0.04, 0.04)	.00	0.19 (0.35)*	0.09	(0.00, 0.37)	.12
Age^b^	0.01 (0.07)	0.02	(-0.04, 0.06)	.01	0.23 (0.41)*	0.09	(0.04, 0.42)	.17	0.02 (0.09)	0.04	(-0.05, 0.09)	.01	-0.02 (-0.11)	0.03	(-0.07, 0.04)	.01	0.24 (0.31)	0.14	(-0.03, 0.52)	.10
Work experience at current preschool^c^	-0.01 (-0.16)	0.01	(-0.03, 0.01)	.03	0.02 (0.07)	0.04	(-0.07, 0.11)	.01	-0.02 (-0.29)	0.01	(-0.05, 0.01)	.08	-0.01 (-0.08)	0.01	(-0.03, 0.02)	.01	-0.02 (-0.06)	0.06	(-0.15, 0.10)	.00
Work experience early childhood education^d^	-0.01 (-0.15)	0.01	(-0.04, 0.02)	.02	0.03 (0.11)	0.06	(-0.08, 0.15)	.01	-0.03 (-0.28)	0.02	(-0.07, 0.01)	.08	-0.01 (-0.14)	0.01	(-0.04, 0.02)	.02	-0.02 (-0.04)	0.08	(-0.17, 0.14)	.00
Support from coworkers	-0.61 (-0.16)	0.71	(-2.07, 0.84)	.02	-1.46 (-0.08)	3.15	(-7.89, 4.98)	.01	0.18 (0.03)	1.08	(-2.02, 2.39)	.00	-0.22 (-0.05)	0.77	(-1.80, 1.35)	.00	-2.11 (-0.09)	4.35	(-11.00, 6.78)	.01
Group work	-0.05 (-0.02)	0.68	(-1.43, 1.32)	.00	2.37 (0.15)	2.93	(-3.61, 8.36)	.02	0.33 (0.06)	1.01	(-1.74, 2.40)	.00	0.11 (0.03)	0.72	(-1.36, 1.59)	.00	2.76 (0.12)	4.06	(-5.53, 11.06)	.02
Innovative climate	0.02 (0.00)	0.70	(-1.40, 1.44)	.00	-1.75 (-0.11)	3.04	(-7.95, 4.45)	.01	1.66 (0.29)	1.00	(-0.38, 3.70)	.09	-0.78 (-0.19)	0.73	(-2.27, 0.71)	.04	-0.85 (-0.04)	4.21	(-9.45, 7.76)	.00

*B* Unstandardized regression coefficients, *β* Standardized regression coefficients, *SE* Standard error

^*^p < .05

^a^Higher educational attainment (% of educators within classrooms with a Bachelor’s or Master’s degree in education)

^b^Younger age (% of educators aged less than 35 years within classrooms)

^c^Less work experience at current preschool (% of educators within classrooms having less than 5 years’ experience)

^d^Less work experience in early childhood education (% of educators within classrooms having less than 10 years’ experience)

It was also investigated whether significant predictors from the univariate analyses (i.e., educators’ educational attainment and age) were significant after adjustment for municipality (Table 4). When examining educators’ educational attainment and municipality simultaneously in the first two models, educational attainment still predicted dose received – exposure (B = 0.15, 95% CI 0.03, 0.27) and the total degree of implementation positively (B = 0.20, 95% CI 0.03, 0.37), and the models were significant. When examining educators’ age and municipality simultaneously in the third model, the model was significant, but age was not significantly associated with dose received – exposure (B = 0.16, 95% C (-0.04, 0.37). No adjusted models were run for the other predictor variables or implementation outcomes since none of them were significant in the univariate analyses.

**Table 4:** Results from the linear regression analyses showing multivariate associations for implementation outcomes in the DAGIS intervention program, 2017–2018 (*N* = 32 preschool classrooms)

	Dose received – exposure			Total degree of implementation		
	B (β)	SE B	(95% CI for B)	B (β)	SE B	(95% CI for B)
Model 1						
Educational attainment^a^	0.15 (0.38)*	0.06	(0.03, 0.27)			
Municipality^b^	8.33 (0.45)**	2.84	(2.53, 14.13)			
Model 2						
Educational attainment^a^				0.20 (0.38)*	0.08	(0.03, 0.37)
Municipality^b^				11.35 (0.44)**	3.94	(3.30, 19.39)
Model 3						
Age^c^	0.16 (0.29)	0.10	(-0.04, 0.37)			
Municipality^b^	5.80 (0.31)	3.23	(-0.80, 12.41)			

Model 1: *p* (model) = .003**; *R*^2^ (adjusted *R*^2^) = .33 (.28); *F*(2, 29) = 6.97

Model 2: *p* (model) = .004**; *R*^2^ (adjusted *R*^2^) = .32 (.27); *F*(2, 29) = 6.77

Model 3: *p* (model) = .015*; *R*^2^ (adjusted *R*^2^) = .25 (.20); *F*(2, 29) = 4.89

*B* Unstandardized regression coefficients, *β* Standardized regression coefficients, *SE* Standard error

^*^p < .05, ** *p* < .01

^a^Higher educational attainment (% of educators within classrooms having a Bachelor’s or Master’s degree in education)

^b^Municipality (1 = Salo, 2 = Riihimäki)

^c^Younger age (% of educators aged less than 35 years within classrooms)

## Discussion

This study examined how various educator factors were associated with implementation of an intervention aimed at promoting healthy EBRBs and self-regulation skills among 3–6-year-old children. The results revealed that classrooms having a higher proportion of educators with a Bachelor’s or Master’s degree in education scored higher on dose received – exposure and their total degree of implementation. The significance of the models was not affected by adjustment for municipality. Moreover, classrooms having a higher proportion of educators younger than 35 years scored higher on dose received – exposure, but this link was non-significant after adjustment for municipality. Educators’ work experience in years and the three dimensions of perceived work environment were not significantly associated with any implementation outcomes.

Our findings indicate that educator’s educational attainment was relevant for some aspects of implementation, mainly the program activities implemented directly by the educators. For example, in a classroom having three educators, having one educator with higher educational attainment resulted on average in an increase of almost five points in dose received – exposure and roughly six points in the total degree of implementation compared to a classroom in which all three educators had low educational attainment. However, the effect size was quite small, as the proportion of explained variance was 12 percent. Our results are somewhat in line with the study by Domitrovich et al. [14], which showed positive associations between teachers’ educational attainment and components of implementation fidelity and suggested that higher educational attainment may provide a better base for teachers to implement content of interventions. Other explanations could be that educators with higher educational attainment are more likely to see the need for health-promoting activities or have better tools for implementing them and/or for overcoming potential barriers. It should also be considered whether intervention materials and/or training sessions are unintentionally better suited for educators with an advanced degree.

Regarding educators’ age, we found that having younger educators (< 35 years) within the classroom was linked to higher dose received – exposure. For example, having one younger educator in a classroom of three educators resulted on average in an increase of more than 8 points in dose received – exposure. In the univariate model, educator’s age was in fact more strongly linked to dose received – exposure than their educational attainment, as the explained variance reached 17 percent. Our finding is partly in line with previous research by Domitrovich et al. [12] showing that teachers younger than 30 years delivered an intervention program of higher quality than older teachers. One suggested mechanism to explain the associations between teachers’ age and implementation outcomes is that younger teachers might be more open to trying new activities in their classrooms and they may be more conscientious about following intervention programs than older teachers [12], although this needs to be explored further in future studies.

In their Consolidated Framework for Implementation Research, Damschroder et al. [34] provided guidance for identifying potential influences on implementation. Among other factors, they suggested that characteristics of the internal organization, such as the quality of social networks and communication within the organization, are important for the successful implementation of interventions. In our study, however, neither of the perceived work environment variables was found to be significantly associated with implementation outcomes. This result may potentially be explained by the fact that educators’ possibility or willingness to implement the DAGIS program may have been affected by other factors unrelated to the personal characteristics of them, such as time resources, adequate staffing in the classrooms, or aspects related to the intervention itself (see Long et al. [35]). It should be noted that the DAGIS program may have been perceived as being quite complex and difficult to implement, as it lasted for approximately 5 months and included more than sixty program activities in addition to the relaxation moments, which were intended to be conducted three times daily.

Our results could be relevant for future health interventions and research. For instance, practitioners seeking to implement health-promoting programs in preschools could recruit classrooms having at least one educator with a university degree or offer additional support or training sessions for classrooms having educators with lower educational attainment and/or educators of older age. Another option could be to enable more adaptation, that is, modifications of the intervention to better fit the contexts in which it is delivered, which might promote program implementation (Harn et al. [36]).

This study has several strengths. Using the framework for process evaluations developed by Saunders et al. [11], several dimensions of implementation on multiple occasions were measured and a diverse set of predictors of variations in implementation was explored. As most previous implementation studies have been conducted in the United States, our study also contributes to the research field with findings from a context outside this region. At the same time, several limitations should be acknowledged. The scoring of the implementation outcomes entailed challenges. As several single items were scored together, we did not consider whether specific components of the dimensions were implemented or not. Also, educators who reported implementing several smaller activities may have achieved approximately the same score as someone who implemented fewer but more time-consuming activities. It is also possible that they reported delivering a higher number of activities but with lower self-reported overall quality, or vice versa, and still scored about the same number of points. The measures in this study and their psychometric properties may also have limitations. Although our measures of implementation were developed using Saunder’s framework, they have not been validated. Our implementation outcomes were also entirely based on self-report measures, rather than observer assessments. For this reason, educators may have overstated their level of implementation. In terms of our measurement of the quality dimension, it might also be difficult for educators to assess how well their classroom received the program. However, similar measures of implementation quality have previously been utilized [18, 22]. There were also some missing values in the logbooks that were interpreted as non-implemented activities, which might have produced biased results since we do not know whether these activities were delivered or why they were not reported. Some values of Cronbach’s alpha were lower for two dimensions of educators’ perceived work environment in the present sample (i.e., support from coworkers = 0.66 and innovative climate = 0.45), which is contrary to earlier studies reporting greater internal consistency [37, 38]. Furthermore, two variables of perceived work environment – support from coworkers and group work – were slightly skewed, potentially affecting the results. This study was also limited by a small sample size, which affected the analyses that we were able to conduct as well as the conclusions that could be drawn. Moreover, individual responses were aggregated into classroom-level variables, which could have affected the results. Using aggregated data can lead to a loss of information at the individual level. The reduction of variability in the data due to aggregation might also lead to inaccurate estimates of standard errors and bias in regression parameters [39, 40]. However, given that the DAGIS intervention was delivered in preschool classrooms by teams consisting of several educators instead of individual implementers, it would not have been appropriate to analyze data at the individual level. Intraclass correlation coefficients were greater than 0.2 for all aggregated variables, indicating an appreciable clustering of individual responses within the preschool classrooms. Another limitation in this study was the fairly small number of responses in each preschool classroom, potentially leading to inaccurate estimations. In addition, some educator data were missing, as 34 of the 145 educators in the original sample did not complete any educator surveys. It is also important to note that most educators in the study had several years of work experience (M = 7.25 years at the current preschool and M = 17.57 years in early childhood education), which might have limited the number of associations that could be detected. Therefore, these findings should be interpreted with caution.

This study advances the line of implementation research by revealing potentially important associations between educator factors and implementation outcomes of an intervention aimed at promoting healthy EBRBs and self-regulation skills among preschool children. More research utilizing larger samples is needed to elucidate how educator factors might facilitate or hinder program implementation. In addition, preschool educators’ actual implementation of intervention programs should be examined in more detail. It would be useful to replicate these findings in other preschool interventions and to explore additional factors (e.g., child and preschool-level characteristics) not assessed in the current study. Moreover, future research should examine whether educator factors are associated with behavioral intervention outcomes among preschool children.

## Conclusions

The findings of this study suggest that preschool educators’ educational attainment and age, as opposed to educators’ work experience in years or perceived work environment in terms of support from coworkers, group work, and innovative climate, might be more relevant for achieving greater implementation levels in interventions targeting children’s EBRBs. Future research should explore whether some educators need additional support and encouragement when delivering these types of intervention programs, or whether intervention materials and/or training sessions are unintentionally better suited for younger educators or educators with an advanced degree.

## Supplementary Information

**Additional file 1.** Dimensions of perceived work environment (QPS_Nordic_).

**Additional file 2.** Items and scoring of dose delivered.

**Additional file 3.** Items and scoring of dose received – exposure.

**Additional file 4.** Items and scoring of dose received – satisfaction.

**Additional file 5.** Items and scoring of perceived quality.

## Data Availability

The datasets used and/or analyzed during the current study are available from the corresponding author on reasonable request.

## Abbreviations

EBRB: Energy balance-related behavior
ICC: Intraclass correlation coefficient
IM: Intervention Mapping
QPSNordic: General Nordic Questionnaire for Psychological and Social Factors at Work

## References

[1] NCD Risk Factor Collaboration (NCD-RisC) (2017). Worldwide trends in body-mass index, underweight, overweight, and obesity from 1975 to 2016: a pooled analysis of 2416 population-based measurement studies in 128·9 million children, adolescents, and adults. Lancet.

[2] Rennie K, Johnson L, Jebb S (2005). Behavioural determinants of obesity. Best Pract Res Clin Endocrinol Metab.

[3] Aparicio E, Canals J, Arija V, De Henauw S, Michels N (2016). The role of emotion regulation in childhood obesity: implications for prevention and treatment. Nutr Res Rev.

[4] Goldfield G, Harvey A, Grattan K, Adamo K (2012). Physical activity promotion in the preschool years: a critical period to intervene. Int J Environ Res Public Health.

[5] Savina E (2020). Self-regulation in preschool and early elementary classrooms: why it is important and how to promote it. Early Childhood Educ J.

[6] Anselma M, Chinapaw M, Kornet-van der Aa D, Altenburg T (2020). Effectiveness and promising behavior change techniques of interventions targeting energy balance related behaviors in children from lower socioeconomic environments: a systematic review. PLoS One.

[7] Becker K, Bradshaw C, Domitrovich C, Ialongo N (2013). Coaching teachers to improve implementation of the good behavior game. Adm Policy Ment Health Ment Health Serv Res.

[8] Pinket A, Van Lippevelde W, De Bourdeaudhuij I, Deforche B, Cardon G, Androutsos O (2016). Effect and process evaluation of a cluster randomized control trial on water intake and beverage consumption in preschoolers from six European countries: the ToyBox-study. PLoS One.

[9] Schaap R, Bessems K, Otten R, Kremers S, van Nassau F (2018). Measuring implementation fidelity of school-based obesity prevention programmes: a systematic review. Int J Behav Nutr Phys Act.

[10] Durlak J, DuPre E (2008). Implementation matters: a review of research on the influence of implementation on program outcomes and the factors affecting implementation. Am J Community Psychol.

[11] Saunders R, Evans M, Joshi P (2005). Developing a process-evaluation plan for assessing health promotion program implementation: a how-to guide. Health Promot Pract.

[12] Domitrovich C, Pas E, Bradshaw C, Becker K, Keperling J, Embry D (2015). Individual and school organizational factors that influence implementation of the PAX good behavior game intervention. Prev Sci.

[13] Dadaczynski K, Hering T (2021). Health promoting schools in Germany. Mapping the implementation of holistic strategies to tackle NCDs and promote health. Int J Environ Res Public Health.

[14] Domitrovich C, Gest S, Gill S, Jones D, DeRousie R (2009). Individual factors associated with professional development training outcomes of the Head Start REDI program. Early Educ Dev.

[15] Bradshaw C, Pas E (2011). A statewide scale up of positive behavioral interventions and supports: a description of the development of systems of support and analysis of adoption and implementation. Sch Psychol Rev.

[16] Driscoll K, Wang L, Mashburn A, Pianta R (2011). Fostering supportive teacher-child relationships: intervention implementation in a state-funded preschool program. Early Educ Dev.

[17] Downer J, Locasale-Crouch J, Hamre B, Pianta R (2009). Teacher characteristics associated with responsiveness and exposure to consultation and online professional development resources. Early Educ Dev.

[18] Ransford CR, Greenberg MT, Domitrovich CE, Small M, Jacobson L (2009). The role of teachers’ psychological experiences and perceptions of curriculum supports on the implementation of a social and emotional learning curriculum. Sch Psychol Rev.

[19] Domitrovich C, Li Y, Mathis E, Greenberg M (2019). Individual and organizational factors associated with teacher self-reported implementation of the PATHS curriculum. J Sch Psychol.

[20] Pas E, Waasdorp T, Bradshaw C (2014). Examining contextual influences on classroom-based implementation of positive behavior support strategies: findings from a randomized controlled effectiveness trial. Prev Sci.

[21] Baker C, Kupersmidt J, Voegler-Lee M, Arnold D, Willoughby M (2010). Predicting teacher participation in a classroom-based, integrated preventive intervention for preschoolers. Early Child Res Q.

[22] Malloy M, Acock A, DuBois D, Vuchinich S, Silverthorn N, Ji P (2014). Teachers’ perceptions of school organizational climate as predictors of dosage and quality of implementation of a social-emotional and character development program. Prev Sci.

[23] Gregory A, Henry D, Schoeny M (2007). School climate and implementation of a preventive intervention. Am J Community Psychol.

[24] Beets M, Flay B, Vuchinich S, Acock A, Li K, Allred C (2008). School climate and teachers’ beliefs and attitudes associated with implementation of the positive action program: a diffusion of innovations model. Prev Sci.

[25] Low S, Van Ryzin M, Brown E, Smith B, Haggerty K (2013). Engagement matters: lessons from assessing classroom implementation of steps to respect: a bullying prevention program over a one-year period. Prev Sci.

[26] Khadjesari Z, Vitoratou S, Sevdalis N, Hull L (2017). Implementation outcome assessment instruments used in physical healthcare settings and their measurement properties: a systematic review protocol. BMJ Open.

[27] Ray C, Kaukonen R, Lehto E, Vepsäläinen H, Sajaniemi N, Erkkola M (2019). Development of the DAGIS intervention study: a preschool-based family-involving study promoting preschoolers’ energy balance-related behaviours and self-regulation skills. BMC Public Health.

[28] Bartholomew Eldredge L, Markham C, Ruiter R, Fernandez M, Kok G, Parcel G (2016). Planning health promotion programs: an intervention mapping approach.

[29] Ray C, Figuereido R, Vepsäläinen H, Lehto R, Pajulahti R, Skaffari E (2020). Effects of the preschool-based family-involving DAGIS intervention program on children’s energy balance-related behaviors and self-regulation skills: a clustered randomized controlled trial. Nutrients.

[30] Maloney JE, Lawlor MS, Shonert-Reichl KA, Whitehead J, Shonert-Reichl KA, Roeser RW (2016). A universal, mindfulness-based social and emotional learning (SEL) Program designed to be implemented in schools by regular classroom teachers. Handbook of mindfulness in education—integrating theory into practice.

[31] Lindström K, Elo A-L, Skogstad A, Dallner M, Gamberale F, Hottinen V (2000). User’s guide for QPSNordic. General Nordic questionnaire for psychological and social factors at work.

[32] Wännström I, Peterson U, Åsberg M, Nygren Å, Gustavsson J (2009). Psychometric properties of scales in the General Nordic Questionnaire for Psychological and Social Factors at Work (QPSNordic): confirmatory factor analysis and prediction of certified long-term sickness absence. Scand J Psychol.

[33] Rubin DB (2004). Multiple imputation for nonresponse in surveys.

[34] Damschroder LJ, Aron DC, Keith RE, Kirsh SR, Alexander JA, Lowery JC (2009). Fostering implementation of health services research findings into practice: a consolidated framework for advancing implementation science. Implement Sci.

[35] Long AC, Hagermoser Sanetti LM, Collier-Meek MA, Gallucci J, Altschaefl M, Kratochwill TR (2016). An exploratory investigation of teachers’ intervention planning and perceived implementation barriers. J Sch Psychol.

[36] Harn BA, Parisi DM, Stoolmiller M (2013). Balancing fidelity with flexibility and fit: what do we really know about fidelity of implementation in schools?. Except Child.

[37] Fløvik L, Knardahl S, Christensen J (2019). The effect of organizational changes on the psychosocial work environment: changes in psychological and social working conditions following organizational changes. Front Psychol.

[38] Himberg-Sundet A, Kristiansen A, Gebremariam M, Moser T, Andersen L, Bjelland M (2019). Exploring the workplace climate and culture in relation to food environment-related factors in Norwegian kindergartens: the BRA-study. PLoS One.

[39] Clark W, Avery K (1976). The effects of data aggregation in statistical analysis. Geogr Anal.

[40] Richter F, Brorsen B (2006). Aggregate versus disaggregate data in measuring school quality. J Prod Anal.

