# Supplementary material for: Role of early childhood educators’ demographic characteristics and perceived work environment in implementation of a preschool health promotion intervention
Source: Arch Public Health. 2023 Jul 7;81:127. doi: 10.1186/s13690-023-01133-z (PMC10326957; doi:10.1186/s13690-023-01133-z)
Supplement: Supplementary file 1 — Additional file 1. Dimensions of perceived work environment (QPSNordic). [file 13690_2023_1133_MOESM1_ESM.docx]

| Dimension | Item |
| --- | --- |
| Support from coworkers | If needed, can you get support and help with your work from your coworkers? |
|  | If needed, are your coworkers willing to listen to your work-related problems? |
| Group work | Do you appreciate belonging to this group or team? |
|  | Is your group or team work flexible? |
|  | Is your group or team successful at problem-solving? |
| Innovative climate | Do workers take initiative at your workplace? |
|  | Are workers encouraged to think of ways to do things better at your workplace? |
|  | Is there sufficient communication in your department? |

Additional file 1. Dimensions of perceived work environment (QPS_Nordic_).
