# Supplementary material for: Role of early childhood educators’ demographic characteristics and perceived work environment in implementation of a preschool health promotion intervention
Source: Arch Public Health. 2023 Jul 7;81:127. doi: 10.1186/s13690-023-01133-z (PMC10326957; doi:10.1186/s13690-023-01133-z)
Supplement: Supplementary file 2 — Additional file 2. Items and scoring of dose delivered. [file 13690_2023_1133_MOESM2_ESM.docx]

Additional file 2. Items and scoring of dose delivered.

| Item / question | Score |
| --- | --- |
| The preschool classroom received the materials and e-mails from the researchers. | 0 = No 7 = Yes |
| What proportion of staff participated in the first training session? | 0 < 25% 1 = 25–49.99% 2 = 50–74.99% 3 = 75–99.99% 4 = 100% ^a^ |
| What proportion of staff participated in the second training session? | 0 < 25% 1 = 25–49.99% 2 = 50–74.99% 3 = 75–99.99% 4 = 100%  ^a^ |
|  | **Maximum total score = 15** |

**^a^** of the preschool classroom.
