# Supplementary material for: Role of early childhood educators’ demographic characteristics and perceived work environment in implementation of a preschool health promotion intervention
Source: Arch Public Health. 2023 Jul 7;81:127. doi: 10.1186/s13690-023-01133-z (PMC10326957; doi:10.1186/s13690-023-01133-z)
Supplement: Supplementary file 3 — Additional file 3. Items and scoring of dose received – exposure. [file 13690_2023_1133_MOESM3_ESM.docx]

Additional file 3. Items and scoring of dose received – exposure.

| Item / question | Response option | Score | |
| --- | --- | --- | --- |
| Number of relaxation moments conducted per day in the preschool classroom | Frequency (*N*) | 0.04 scores for each relaxation moment   10.8 = Maximum total score for all conducted relaxation moments during the intervention (maximum 270 sessions) | |
| **Theme period: Strengthening self-regulation skills** |  |  | |
| Did the educators of the classroom deliver the materials to parents? | No / Yes | 0 = No 0.5 = Yes | |
| Has your preschool classroom implemented the following recommended tasks / activities so far? |  |  | |
| Crafted 'The Wise Owl' and 'The Watchdog' [fictive animal characters]? | No / Yes | 0 = No 1 = Yes |  |
| Crafted 'The Treasure Chest'? | No / Yes | 0 = No 1 = Yes |  |
| Crafted 'The Watchdog Calming down jar'? | No / Yes | 0 = No 1 = Yes |  |
| Integrated themes and characters into everyday situations (e.g., discussions about the child's own actions in everyday situations with the help of characters)? | Never / Once / Several times | 0 = Never 0.5 = At least once |  |
| Collected 'Diamonds of success' in 'The Treasure Chests'? | Never / Once / Several times | 0 = Never 0.5 = At least once |  |
| Conducted exercises of mindful attention? | Never / Once / Several times | 0 = Never 0.5 = At least once |  |
| Utilized the supplementary materials (e.g., storybooks, non-fiction books, discussions, games) ? | Never / Once / Several times | 0 = Never 0.5 = At least once |  |
| Developed and utilized own supplementary materials? | Never / Once / Several times | 0 = Never 0.5 = At least once |  |
| **Theme period: Enhancing physical activity** |  |  | |
| Did the educators of the classroom deliver the materials to parents? | No / Yes | 0 = No 0.5 = Yes | |
| Did the preschool classroom arrange the activity afternoon? | No / Yes | 0 = No 2 = Yes | |
| Has your preschool classroom implemented the following recommended tasks / activities so far? |  |  | |
| Read the storybook to the children? | No / Yes | 0 = No 1 = Yes | |
| Crafted the 'Cinnamon characters' [fictive animal characters]? | No / Yes | 0 = No 1 = Yes | |
| Encouraged children to take the 'Cinnamon character' home? | No / Yes | 0 = No 1 = Yes | |
| Taken photos of and held an exhibition of the 'Cinnamon characters' on the DAGIS wall? | No / Yes | 0 = No 1 = Yes | |
| Conducted the physically active Christmas calendar? | No / Yes | 0 = No 1 = Yes | |
| Processed the content of the storybook through discussion or in any other way? | Never / Once / Several times | 0 = Never 0.5 = At least once | |
| Included the crafted 'Cinnamon character' or the other characters in other activities (e.g., showing things to do outdoors)? | Never / Once / Several times | 0 = Never 0.5 = At least once | |
| Collected 'Diamonds of success' in the 'Treasure Chests'? | Never / Once / Several times | 0 = Never 0.5 = At least once | |
| Played games in which children focused on listening to sounds and instructions? | Never / Once / Several times | 0 = Never 0.5 = At least once | |
| Played exercise games in which children practiced mindful watching? | Never / Once / Several times | 0 = Never 0.5 = At least once | |
| Utilized the supplementary materials (e.g., exercises focused on discussion, vision, relaxation, attention, or memory)? | Never / Once / Several times | 0 = Never 0.5 = At least once | |
| Developed and utilized own supplementary materials? | Never / Once / Several times | 0 = Never 0.5 = At least once | |
| Was the feedback on children's physical activity visible on the preschool wall or bulletin board? | No / Yes | 0 = No 1 = Yes | |
| **Theme period: Promoting consumption of fruits and vegetables** |  |  | |
| Did the educators of the classroom deliver the materials to parents? | No / Yes | 0 = No 0.5 = Yes | |
| Did the preschool classroom arrange the activity afternoon? | No / Yes | 0 = No 2 = Yes | |
| Has your preschool classroom implemented the following recommended tasks / activities so far? |  |  | |
| Read the storybook to the children? | No / Yes | 0 = No 1 = Yes | |
| Crafted the 'Star eye characters' [fictive animal characters]? | No / Yes | 0 = No 1 = Yes | |
| Encouraged children to take the 'Star eye character' home? | No / Yes | 0 = No 1 = Yes | |
| Taken photos of and held an exhibition of the 'Star eye characters' on the DAGIS wall? | No / Yes | 0 = No 1 = Yes | |
| Processed the content of the storybook by discussion or in any other way? | Never / Once / Several times | 0 = Never 0.5 = At least once | |
| Included the crafted 'Star eye character' or the other characters in other activities (e.g., in eating activities)? | Never / Once / Several times | 0 = Never 0.5 = At least once | |
| Collected 'Diamonds of success' in the 'Treasure Chests'? | Never / Once / Several times | 0 = Never 0.5 = At least once | |
| Conducted smelling exercises (together with the 'Star Eye character')? | Never / Once / Several times | 0 = Never 0.5 = At least once | |
| Conducted exercises involving tasting and toucing food (together with the 'Star Eye character')? | Never / Once / Several times | 0 = Never 0.5 = At least once | |
| Utilized the supplementary materials (e.g., exercises focused on discussion, vision, relaxation, attention, or memory)? | Never / Once / Several times | 0 = Never 0.5 = At least once | |
| Developed and utilized own supplementary materials? | Never / Once / Several times | 0 = Never 0.5 = At least once | |
| **Theme period: Reducing excessive screen time** |  |  | |
| Did the educators of the classroom deliver the materials to parents? | No / Yes | 0 = No 0.5 = Yes | |
| Did the preschool classroom arrange the activity afternoon? | No / Yes | 0 = No 2 = Yes | |
| Has your preschool classroom implemented the following recommended tasks / activities so far? |  |  | |
| Re-read the storybook of the 'Cinnamon character'? | No / Yes | 0 = No 1 = Yes | |
| Brought up the screen theme in conjunction with the “living in the old days” -theme? | No / Yes | 0 = No 1 = Yes | |
| Used the DAGIS wall? | No / Yes | 0 = No 1 = Yes | |
| Used the 'Wise owl' map application? | Never / Once / Several times | 0 = Never 0.5 = At least once | |
| Discussed the 'Wise owl' map application with parents? | Never / Once / Several times | 0 = Never 0.5 = At least once | |
| Conducted mindful movement exercises? | Never / Once / Several times | 0 = Never 0.5 = At least once | |
| Collected 'Diamonds of success' in the 'Treasure Chests'? | Never / Once / Several times | 0 = Never 0.5 = At least once | |
| Integrated the characters and themes into everyday situations? | Never / Once / Several times | 0 = Never 0.5 = At least once | |
| Utilized the supplementary materials? | Never / Once / Several times | 0 = Never 0.5 = At least once | |
| Developed and utilized own supplementary materials? | Never / Once / Several times | 0 = Never 0.5 = At least once | |
| Was the feedback on children's screen time visible on the preschool wall or bulletin board? | No / Yes | 0 = No 1 = Yes | |
| **Theme period: Restricting the consumption of sugary foods and beverages** |  |  | |
| Did the educators of the classroom deliver the materials to parents? | No / Yes | 0 = No 0.5 = Yes | |
| Did the preschool classroom arrange the activity afternoon? | No / Yes | 0 = No 2 = Yes | |
| Has your preschool classroom implemented the following recommended tasks / activities so far? |  |  | |
| Re-read the storybook of the 'Star eye character'? | No / Yes | 0 = No 1 = Yes | |
| Arranged the 'Wise owl's fruit and veggie potluck'? | No / Yes | 0 = No 1 = Yes | |
| Used the DAGIS wall? | No / Yes | 0 = No 1 = Yes | |
| Expressed / classified emotional states (optimism, pessimism)? | No / Yes | 0 = No 1 = Yes | |
| Grown edible Easter grass and discussed growth and having confidence in the future? | No / Yes | 0 = No 1 = Yes | |
| Glued 'Diamonds of success' to the 'I can and I succeed' -self-portraits? | No / Yes | 0 = No 1 = Yes | |
| Positive food talk, noticing strengths and skills in eating? | Never / Once / Several times | 0 = Never 0.5 = At least once | |
| Integrated the characters and themes into everyday situations? | Never / Once / Several times | 0 = Never 0.5 = At least once | |
| Collected 'Diamonds of success' in the 'Treasure Chests'? | Never / Once / Several times | 0 = Never 0.5 = At least once | |
| Utilized the supplementary materials? | Never / Once / Several times | 0 = Never 0.5 = At least once | |
| Developed and utilized own supplementary materials? | Never / Once / Several times | 0 = Never 0.5 = At least once | |
|  |  | **Maximum total score = 59.8** | |
|  | Adjustment of the total score in proportion to the other implementation outcomes (x 0.75) | **Final total score (0.75 x 59.8) = 44.85** | |
