# Supplementary material for: Role of early childhood educators’ demographic characteristics and perceived work environment in implementation of a preschool health promotion intervention
Source: Arch Public Health. 2023 Jul 7;81:127. doi: 10.1186/s13690-023-01133-z (PMC10326957; doi:10.1186/s13690-023-01133-z)
Supplement: Supplementary file 4 — Additional file 4. Items and scoring of dose received – satisfaction. [file 13690_2023_1133_MOESM4_ESM.docx]

Additional file 4. Items and scoring of dose received – satisfaction.

| Item / question | Response option | Score |
| --- | --- | --- |
| What do you think of the DAGIS program (content and activities) in general? | 1 (I did not like it at all) - 5 (I liked it a lot) | 2 = classroom mean value 1 < 1.99 4 = classroom mean value 2 < 2.99 6 = classroom mean value 3 < 3.99 8 = classroom mean value 4 < 4.99 10 = classroom mean value 5 |
| The DAGIS program was a good fit for our classroom. | 1 (Totally disagree) -  5 (Totally agree) | 0 = classroom mean value < 4 0.5 = classroom mean value > 4 |
| The DAGIS program was easy to incorporate into the activities of the preschool classroom. | 1 (Totally disagree) -  5 (Totally agree) | 0 = classroom mean value < 4 0.5 = classroom mean value > 4 |
| The DAGIS program caused a lot of extra work. | 1 (Totally disagree) –  5 (Totally agree) | 0 = classroom mean value > 2 0.5 = classroom mean value < 2 |
| The DAGIS program was not flexible. | 1 (Totally disagree) –  5 (Totally agree) | 0 = classroom mean value > 2 0.5 = classroom mean value < 2 |
| The DAGIS program included too many components to be implemented. | 1 (Totally disagree) –  5 (Totally agree) | 0 = classroom mean value > 2 0.5 = classroom mean value < 2 |
| The DAGIS program lasted too long. | 1 (Totally disagree) –  5 (Totally agree) | 0 = classroom mean value > 2 0.5 = classroom mean value < 2 |
| I found that the DAGIS program's activities and materials regarding self-regulation and calming techniques were useful to me. | 1 (Totally disagree) –  5 (Totally agree) | 0 = classroom mean value < 4 0.5 = classroom mean value > 4 |
| I found that the DAGIS program's activities and materials regarding self-regulation and calming techniques included fun and inspiring activities for children. | 1 (Totally disagree) –  5 (Totally agree) | 0 = classroom mean value < 4 0.5 = classroom mean value > 4 |
| I found that the DAGIS program's activities and materials regarding physical activity and screen use were useful to me. | 1 (Totally disagree) –  5 (Totally agree) | 0 = classroom mean value < 4 0.5 = classroom mean value > 4 |
| I found that the DAGIS program's activities and materials regarding physical activity and screen use included fun and inspiring activities for children. | 1 (Totally disagree) –  5 (Totally agree) | 0 = classroom mean value < 4 0.5 = classroom mean value > 4 |
| I found that the DAGIS program's activities and materials regarding consumption of vegetables, fruits, and sugar in the DAGIS program were useful to me. | 1 (Totally disagree) –  5 (Totally agree) | 0 = classroom mean value < 4 0.5 = classroom mean value > 4 |
| I found that the DAGIS program's activities and materials regarding consumption of vegetables, fruits, and sugar included fun and inspiring activities for children. | 1 (Totally disagree) –  5 (Totally agree) | 0 = classroom mean value < 4 0.5 = classroom mean value > 4 |
|  |  | **Maximum total score = 16** |
