# Supplementary material for: Role of early childhood educators’ demographic characteristics and perceived work environment in implementation of a preschool health promotion intervention
Source: Arch Public Health. 2023 Jul 7;81:127. doi: 10.1186/s13690-023-01133-z (PMC10326957; doi:10.1186/s13690-023-01133-z)
Supplement: Supplementary file 5 — Additional file 5. Items and scoring of perceived quality. [file 13690_2023_1133_MOESM5_ESM.docx]

Additional file 5. Items and scoring of perceived quality.

| Item / question | Response option | Score |
| --- | --- | --- |
| Overall, how well do you think your classroom delivered the DAGIS program (content and activities)? | 1 (Very poorly) –  10 (Very well) | 1.5 = classroom mean value 1 < 1.99 3 = classroom mean value 2 < 2.99 4.5 = classroom mean value 3 < 3.99 6 = classroom mean value 4 < 4.99 7.5 = classroom mean value 5 < 5.99 9 = classroom mean value 6 < 6.99  10.5 = classroom mean value 7 < 7.99  12 = classroom mean value 8 < 8.99 13.5 = classroom mean value 9 < 9.99 15 = classroom mean value 10 |
|  |  | **Maximum total score = 15** |
